# Rohbau3D: A Shell Construction Site 3D Point Cloud Dataset

**DOI:** 10.1038/s41597-025-05827-7

**Published:** 2025-08-25

**Authors:** Lukas Rauch, Thomas Braml

**Affiliations:** Institute of Structural Engineering, University of the Bundeswehr Munich, Munich, Germany

**Keywords:** Civil engineering, Architecture

## Abstract

We introduce Rohbau3D, a novel dataset of 3D point clouds that realistically represents indoor construction environments. The dataset comprises 504 high-resolution LiDAR scans captured with a terrestrial laser scanner across 14 distinct construction sites from high-rise buildings, all in various stages of shell construction or under renovation. Each point cloud is enriched with the scalar laser reflectance intensity, RGB color values, and reconstructed surface normal vectors. In addition to the 3D data, the dataset includes high-resolution 2D panoramic renderings of each scene and their associated point cloud features. Designed to reflect the complexity and variability of construction site data and released to address the shortage of real-world data for geometric processing in construction applications, Rohbau3D facilitates research into scene understanding and computer vision for structural and civil engineering. To our knowledge, it is the first dataset of its kind and scale to be published. Rohbau3D is designed as a foundation for ongoing work, with intention to extend it through additional targeted annotations and as a benchmark to support future research.

## Background & Summary

**Rohbau** [ˈroːbaʊ] is the German term for the stage in a building project where the basic structural framework—the shell of the building—is completed. A construction in this phase includes the foundation, load-bearing walls, floors, and the roof framework. At this point, the building has taken shape externally, but the interior remains unfinished: insulation, plastering, flooring, and the installation of windows, doors, electrical, and plumbing systems are yet to be done. Shell construction sites are inherently harsh environments, shaped by rough terrain, continuously evolving workspaces, and weather-dependent lighting conditions. These factors create operational challenges on site for robotic systems with optical sensors, and the corrupted data from such environments can degrade results in downstream processing tasks.

In civil engineering, the acquisition and manual evaluation of laser-scanned point clouds for the reconstruction of digital models is considered standard practice, facilitating the extraction of precise geometric as-built information. Terrestrial laser scanners (TLS) are particularly popular within this procedure due to their ease of use, high measurement accuracy, and the customary integration of domain-specific interfaces to share measurements with computer-aided design (CAD), drafting software, and project planning tools. Despite this, the degree of automation based on TLS-derived data remains low. Data handling and evaluation are largely performed manually by trained specialists, since the variability and complexity of site conditions demand human flexibility and contextual understanding. This has led to increasing demand for methods that simplify digitization and enable efficient processing of large-scale data.

Empirically, the use of a consistent 3D model for planning and construction is only possible for large prestige projects where the client bears the additional costs of creating a Building Information Model (BIM). In contrast, small and medium-sized projects—which make up most of the built environment— continue to be planned and implemented using 2D plans. Architectural 3D models, if used at all, often serve only for client visualization and are not suitable for object-specific digital information exchange^1,2^. The gap is even more pronounced in the renovation, preservation, and repurposing of existing buildings, for which no digital model of the original structure has ever existed.

To create new research directions for modern engineering disciplines, practitioners need to understand the characteristics of geometric data and overcome the challenges associated with their surveying and processing. In the fields of 3D computer vision, spatial perception, and reconstruction, deep learning models are now capable of processing sufficiently large datasets and generalizing even in challenging environments. While several large datasets exist that capture spatial scenarios from autonomous driving^3–5^, infrastructure^6,7^, and the interior of mixed-use buildings^8,9^, comparable data addressing construction site environments are insufficient^10^.

To fill this gap, with Rohbau3D^11^, we present a novel dataset utilizing 3D point cloud captures of indoor environments in the final shell construction phases. The dataset consists of 500 + point clouds from a wide range of scenarios including multiple building types and utilization classes, a variety of architecture and building techniques, and object sizes ranging from small residential housing to multi-story public buildings. Every data point in this data repository consists of the bare point cloud coordinates, the reflection intensity of the laser sensor, the RGB colorization, and a reconstruction of the per-point surface normal vector. In extent to the point cloud data, the repository also includes a high-resolution panorama rendering of the scene and its point cloud features.

**Fig. 1:**
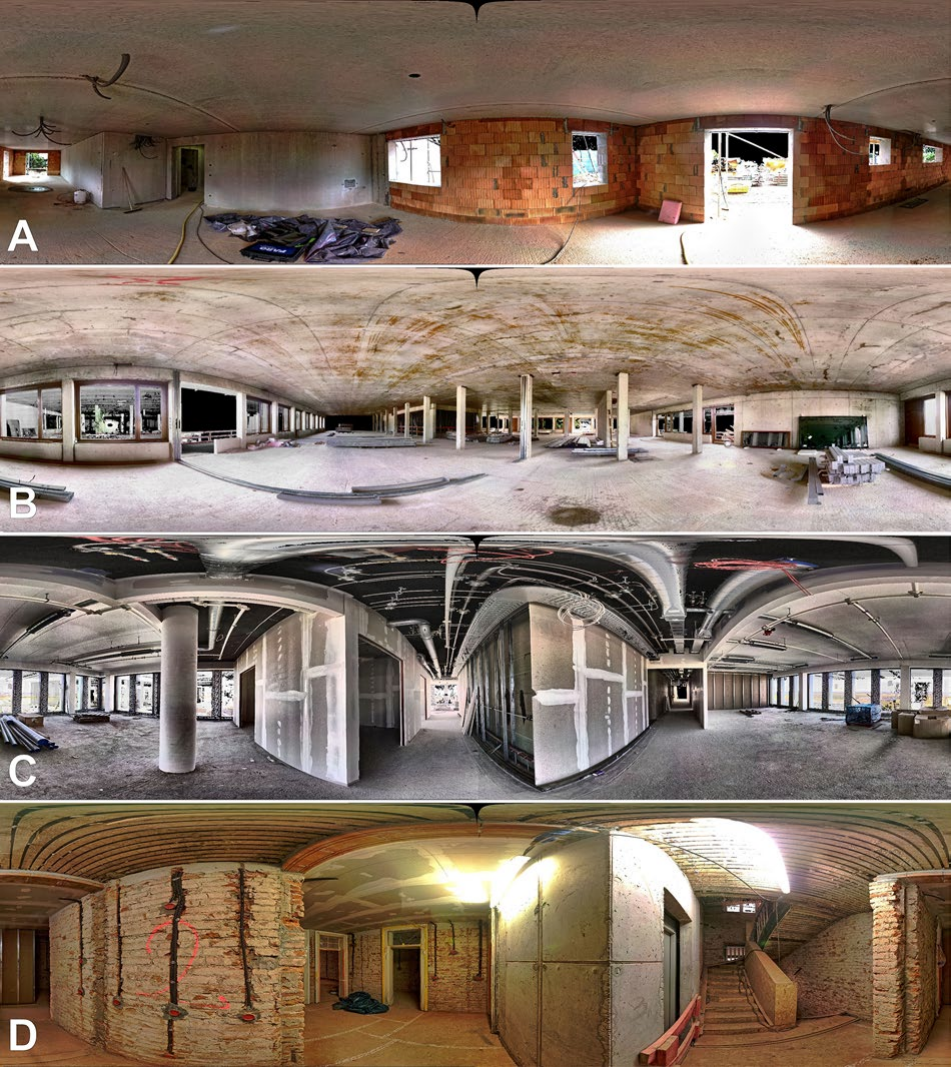
Panoramic renderings of four exemplary scenes from the Rohbau3D dataset. (**A**) New construction of an urban multi-apartment block in brick and reinforced concrete construction. (**B**) New construction of a multi-story school building in steel-skeleton construction with large room layouts. (**C**) New construction of a high-rise office block with pre-installed technical building equipment and drywall partitioning. (**D**) Core refurbishment of an old existing residential building with exposed brick walls and historic ceiling structure.

The architecture of all buildings is described as modern central European style and all construction sites are located in a one-hour radius around the city of Munich, Germany. The residential buildings are predominantly combined brick construction with prefabricated reinforced concrete elements. The refurbished buildings show considerable damage to the core structure and rudimentary condition of the building shell in some scenes. The educational facilities and the office building are all built in classic reinforced concrete skeleton construction, which is subsequently subdivided into smaller units using drywall construction. Panoramic point cloud to image renderings of the different building environments are visualized in Fig. 1.

At the time of data acquisition, all construction sites were in the shell construction stage. External contours, load-bearing components such as walls and columns and the roof structure had already been completed. Among other things, the windows and facade paneling are often missing, and the interior work has not yet been completed at this early stage of construction.

### Related work

Early 3D datasets of indoor spaces are frequently captured with commodity short-range depth scanners, however comprising RGB-D sequences with low resolution and a limited field of view^12,13^. SceneNN^14^ and ScanNet^15^ provide 3D reconstructions from RGB-D streams with labelled triangle meshes of apartment buildings, although the rooms are often incompletely mapped, and the quality of the recordings is limited by the handheld devices. Higher resolution and full 3D coverage are achieved by tripod-mounted RGB and structured light camera rigs. Matterport3D^16^ generates textured meshes from panoptic RGB-D images of indoor home environments, while the S3DIS benchmark^17^ generates similar data from the interior of educational and office buildings. The colored 3D point clouds in Matterport3D and S3DIS are sampled uniformly from textured meshes and do not represent the unique scan pattern of laser scans. ScanNet++^18^ couples high-quality terrestrial laser scan with DSLR camera images and commodity iPhone RGB-D frames for novel view synthesis in the interiors of a university, resulting in richly annotated Poisson mesh reconstructions of indoor environments. In addition to these mesh-based reconstructions, LiDAR-Net^19^ provides a dataset with raw LiDAR point clouds with annotations from indoor areas of everyday life. The data is characterized by its non-uniform point distribution (scanning holes, scanning lines) and anomalies (reflection artifacts, ghosting). All these datasets address the issue of understanding interior scenes with only a limited focus on the building structure itself. Technically, shell construction sites are also interior spaces, but furnished and inhabited spaces cannot be compared to the unique environment during construction phase.

Taking into account a fourth dimension (time), the Hilti SLAM Challenge^20^ provides mobile LiDAR data from three different multi-story construction sites and the required ground truth data to benchmark simultaneous localization and mapping (SLAM) techniques in construction environments. The “Nothing Stands Still” benchmark^21^ addresses more radical change in the structure of the built environment for spatiotemporal registration tasks and publishes a point cloud dataset that focuses on interior layouts, where the exterior shell of the building has been erected, and the interior space is empty. The captures chronicle the progression of various construction activities. The ConSLAM dataset^22^ can be placed thematically in the middle between the other two. The dataset consists of four sequences, captured within the course of four months on the same floor of a construction site, to evaluate SLAM accuracy for construction site progress monitoring and quality control systems. All three datasets are designed for specific use cases of mapping and registration.

Synthetic data addresses the demand for more resources in extension to real-world datasets, including the built environment. Virtual image and LiDAR data from driving within a computer game^23^, synthetic mobile laser scans from virtual urban and suburban environments^24^, and synthetic point clouds from the CARLA simulator to reproduce real-world street data^6^ have shown to be valuable for research in autonomous driving. For the virtual representation of indoor spaces, resources exist in the format of building information models (BIM) where annotations are derived directly from the BIM component names and synthetic point clouds can be sampled from simulated laser scanning^25,26^, or in the format of photo-realistic RGB-D image renderings with segmentation masks, which can also be used to project image pixels into 3D point clouds^9,27^. Synthetic data is a way of creating versatile scenarios and scaling data generation to provide volumes of data that cannot be realized with traditional approaches. However, real data will always be essential to validate and test real-world performance.

Our proposed dataset differs from the previous work in terms of the content of the data depicted and the data realism. Rohbau3D is the first collection of its kind to document versatile scenes from high-rise building construction sites in high resolution, collected with a professional-grade terrestrial laser scanner, in comparison to mobile lidar scanners with low-resolution and lower measuring accuracy^19,20^. The ConSLAM dataset^22^ is comparable in terms of data acquisition, but it shows the same building in all four sequences to document the changes over time. Rohbau3D, on the other hand, showcases the diversity of 14 different construction sites. As the point clouds in Rohbau3D were not sampled from surface meshes, the data more accurately captures reality. Laser scans are always contaminated by reflections, noise and ghost points due to imperfect measurements and the physics of light. These phenomena can only be reproduced with real data and cannot be simulated.

## Methods

### Data acquisition

The data used for our dataset was collected over the course of a year in and around the city of Munich, Germany. The specific construction sites were jointly selected and provided by four different construction companies, ranging from small businesses to a large corporation and the Munich State Building Authority, to achieve wide coverage and object diversity.

The individual scenes vary in light conditions, surface finishes (e.g., brick walls, concrete, plaster, drywall), and the levels of contamination. In some cases, the environment in the scenes is very tidy, in others, it is characterized by disruptive elements such as building rubble, stored building materials or scaffolding for structural support, which partially obscures the view. Typical for laser scans, the dataset contains various challenging elements, such as reflection artifacts from mirroring surfaces (e.g., wet spots) and transparent objects (e.g., glass windows).

**Fig. 2:**
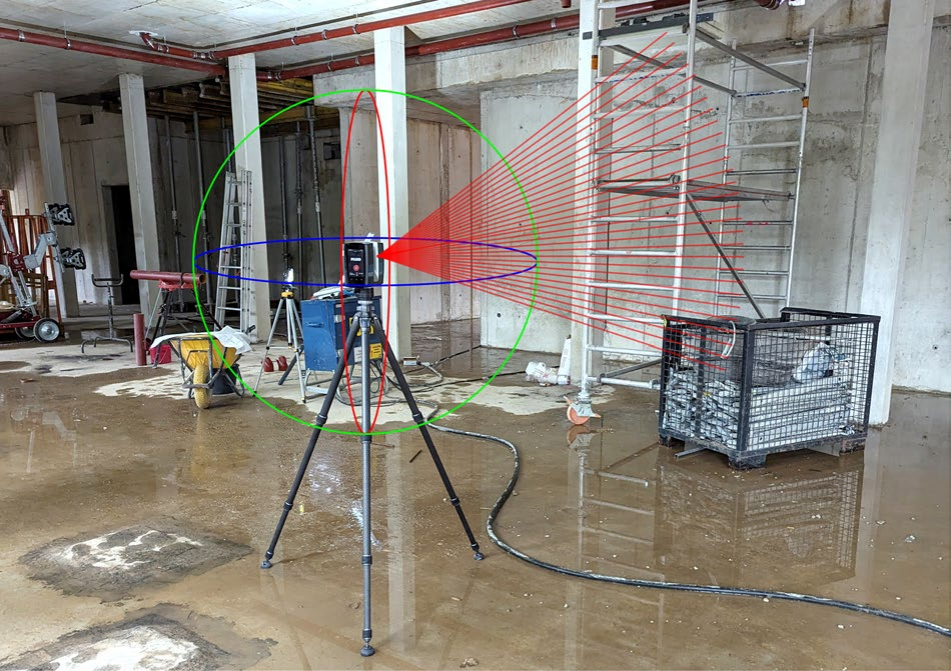
Exemplary illustration of the terrestrial laser scanner device that was used to record the data. The laser scans the room in a spherical grid. The scene in the background shows an example of a particularly challenging situation on the construction site, which presents difficulties for the optical measurement method due to the confusing scene, the building materials, tools and temporary support structures stored on the construction site, and puddles of water on the ground, which irritate the laser reflection.

As illustrated in Fig. 2, all point clouds were captured using the FARO Focus M70 terrestrial laser scanner, which scans up to 1 million spherical points per second by LiDAR technology and can produce colored point clouds by superimposing spherical high dynamic range (HDR) images in post-processing. Comparable business-grade laser scanners are used in construction management and supervision to measure, document, and evaluate ongoing construction processes. The laser scanner device is tripod mounted, which improves the measurement quality in contrast to handheld devices. The selection of scan positions does not follow any strict rules but is adapted to the respective situation by a trained civil engineer on site, in a way that a minimum number of scan positions can be used to achieve maximum coverage.

Compared to mobile LiDAR systems, the terrestrial scanner produces a very high point density, high 3D accuracy, and low noise instead of irregular scan patterns. On average, every scan consists of 2.34e + 07 points (min = 2.73e + 06, max = 4.28e + 07, std.dev. =  ±3.38e + 06), with a mean surface density of 31.7 points/cm^2^ (std.dev. =  ±15.4), and a mean volume density of 9.56 points/cm^3^ (std.dev. =  ±4.62) calculated based on nearest neighboring points in a radius = 10 cm spherical neighborhood. The high resolution of the TLS data and the availability of the color information is a key difference, and a decisive added value to point cloud data created with mobile LiDAR systems.

The individual scans per site are not registered in a shared coordinate frame. For each point set, the scanner’s center position is placed at the coordinate origin (0, 0, 0) and all point coordinates are therefore distributed around that pose. As the sensor origin is in the x-y plane, measuring points below the sensor head, e.g. the floor slab, have a negative sign of the z-coordinate. The geographical orientation around the z-axis in individual scans does not follow a fixed, cardinal direction, appearing quasi-random from the unique device placement. This ensures that each scan in the dataset is standardized and has the same formal prerequisites.

Point cloud preprocessing was kept to a minimum. The only manipulation of the raw data has involved applying a Euclidean distance filter to exclude points outside a sphere of radius 25 meters from the coordinate origin. This filter reduces the number of faulty measurements, often caused by highly reflective surfaces or other sensor disturbances, which otherwise add no value to the analysis and may even corrupt it. The accuracy of the 3D position decreases significantly at greater distances. The manufacturer quantifies the 3D accuracy as 2 mm at 10 m, 3 mm at 25 m and an added uncertainty of 0.1 mm/m for distances above^28^. Besides the accuracy, the surface sample density decreases at greater distances, which has a negative effect on the preservation of smaller shape details.

### Point cloud features

**Fig. 3:**
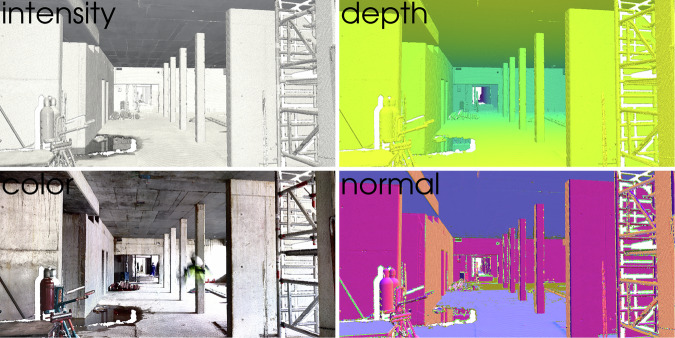
Overview of the point cloud features included in the Rohbau3D dataset.

Together with the coordinates that represent the geometry of the scenes, the dataset includes additional features that annotate the coordinate points with semantic information. A representation of the point cloud features in the dataset is given in Fig. 3. The LiDAR sensor can detect surfaces by the time a light impulse takes to bounce back from a reflective object. At the same time, the sensor measures the intensity of the returning near-infrared light pulse and thus characterizes the reflectivity of a sensed object. Scanning the surroundings is spherical, but the released dataset describes geometries in terms of its x, y, and z coordinates and in line with most geometry data types. However, since the viewpoint is known, the polar coordinate representation $(r,\,\theta ,\,\phi )$ can be reconstructed using a simple coordinate transformation^29^, as displayed in the formulas below:

1 $$r=\sqrt{{x}^{2}+{y}^{2}+{z}^{2}}$$

2 $$\theta ={\tan }^{-1}\left(\frac{\sqrt{{x}^{2}+{y}^{2}}}{z}\right)$$

3 $$\phi ={\tan }^{-1}\left(\frac{y}{x}\right)$$

From the polar coordinate representation, the point distance to the origin can be derived to generate a metric depth feature map. The reflection intensity is given as a per-point scalar feature vector. According to the ASTM E57 file format for 3D imaging data exchange, the reflection intensity $I$ is encoded as a normalized value ($I\in [\mathrm{0,1}]$) by the intensity limits of a sensor device^30^. Poorly reflective surfaces appear at the lower end of the scale, close to zero, and are displayed in a correspondingly dark color. Highly reflective surfaces are displayed close to one and are bright. The factors influencing intensity include range, surface normal, surface color, surface material, and humidity, among others. The scalar intensity can prove to be valuable in construction-related computer vision applications as multiple studies report improvements in material segmentation by utilizing the spectral information of near-infrared (NIR)^31–34^.

The LiDAR sensor itself cannot detect color information, even though the color of a surface influences its reflectivity. To generate a colored point cloud output, the FARO Focus laser scanner maps HDR image data from a subsequent acquisition process. One disadvantage of this method is that the capturing of photographs and LiDAR data cannot be processed simultaneously. This induces a shift in the timestamp of color and geometry data and can produce color artifacts in rapidly changing or dynamic environments. Nevertheless, the fundamental structure of buildings remains static, and the effect is therefore negligible in most cases. RGB color information helps to contextualize the point cloud because of its familiar resemblance to 3D photographs. Surface normal vectors are descriptors of the local geometric features that help to understand the nature of an underlying surface. In computer graphics, the normal vector and its orientation is a prerequisite for surface reconstruction, scene lighting and other visual effects. These local features cannot be obtained directly from the laser measurements, instead the normal vectors at each measurement point were reconstructed using learnt hyper surfaces^35^. The global normal’s orientation in all scenes is uniformly defined inwards in the direction of the viewpoint.

### Surface normal reconstruction

In spatial scanning with laser pulses, the sensor measures the travelling time of the light pulse in order to calculate the object distance and the intensity of the reflected light. The orientation of the reflecting surface cannot be deduced from the measurement data, although the angle of reflection has a proportionate influence on the intensity of the reflected return. As mentioned above, the intensity of reflection return depends, in general, upon the distance to the object, the angle between the surface normal and the incident laser ray and the glossiness of the surface^36^.

For the subsequent reconstruction of the surface normals from unstructured point clouds in Rohbau3D, we deployed the learning-based method according to Li *et al*.^35^. We have come to this decision after comparing the normal estimation accuracy of various learning and non-learning approaches on different benchmarks under consideration of the oriented and unoriented error. The validation of this method is discussed in the Technical Validations section. We extend the original reconstruction model with modifications that enable processing of very large TLS point clouds, containing several million points per scan. The model learns signed hyper surfaces (SHS) in a high-dimensional feature space, where local and global information is arranged to estimate oriented normals from point clouds in an end-to-end manner. The model pipeline consists of two parallel branches, i.e., the local patch encoding and the global shape encoding, whose joint output is used as latent input for learning the signed hyper surface in the feature space. Finally, an embedding of the signed hyper surface was used to regress the oriented normal of the query point. A local patch ${{\boldsymbol{P}}}_{q}$ of the point cloud ${\mathscr{P}}$ was used to capture the local geometry for accurately describing the surface around a query point $q$. Since the interior/exterior of a surface cannot be determined reliably from a local patch, a global subsample ${{\boldsymbol{P}}}_{q}$ was extracted from the point cloud ${\mathscr{P}}$ to provide additional information for sign estimation at point *p*^35^.

The model was trained on the general PCPNet dataset^37^, which is curated specifically for the purpose of learning local shape properties, and thus includes a variety of sharp corners, detail-rich geometric features, extended flat surfaces, and smooth curvatures. The fact that the shapes in the training dataset differ in size and appearance from the building scenes in Rohbau3D is irrelevant, as each patch within the model is nationalized to the size of a unit sphere. Differences in size can be generalized by scaling, but the difference in point-sample density with respect to actual scan data remains an issue.

**Fig. 4:**
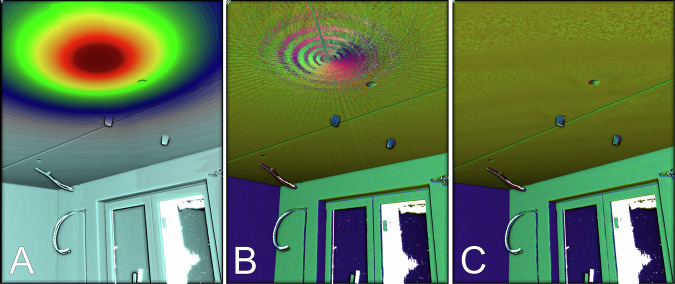
(**A**) Volumetric point cloud density distribution on scanned surfaces. dark red = high density; light blue = low density. (**B**) Without subsampling the global patch encoder input, predicted normals show disturbance in areas of high point density. (**C**) With subsampling the global patch encoder input, predicted normals show no disturbance in areas of high point density.

We found that in certain areas of the point cloud, where the density is extremely high, the estimated results degrade. For indoor locations with low ceilings, this is always the case directly above the laser scanner due to TLS’s rotational sensing pattern. Figure 4(A) shows the volume density distribution above the device for an indoor scan, and Fig. 4(B) the disturbance of predicted normal vectors in this area. Our solution to that is to use point discretization prior to the model’s patch encoding. By default, SHS-Net deploys a k-dimensional tree (k-d tree) to find the $k$ nearest neighbors (knn) around a query point $q$ to sample a local patch ${{\boldsymbol{p}}}_{q}$. The default neighborhood size is set to 700. In regions of high point density, this neighborhood has a small expansion and the selected *k* neighbors lie approximately in a singular point, too close to capture the local geometry. Subsampling the point cloud ${\mathscr{P}}$ using a discrete voxel grid $\hat{{\mathscr{P}}}$ with fixed voxel size of 1 × 1 × 1 cm helps to build a more evenly distributed local patch ${{\boldsymbol{p}}}_{q}$ in the presence of varying point density. The smaller size of the k-d tree also has the advantage that the computation of the knn search is faster, which overall makes a noticeable difference for very large scenes. The difference in the results is visualized in the Fig. 4(C), in which the interference is no longer present.

Similarly to the subsampling of the patch encoder input, we also discretized the shape encoder input to account for the sheer scene size of our real-world laser scans. The algorithm requires the model to parse each point in the scene individually, by sampling the local and global patch, to finally infer the predicted normal. We found that the sequential patch sampling, where the global patch ${{\boldsymbol{P}}}_{q}$ is drawn, is the decisive bottleneck for the overall inference pipeline. In SHS-Net, a probability-based sampling strategy^38^ is used to obtain ${{\boldsymbol{P}}}_{q}$, which statistically brings more points closer to the query point $q$. It samples points according to a density gradient that decreases with increasing distance from the point *q*^35^. However, the calculation of the distance vector for the full point cloud ${\mathscr{P}}$ is computationally intensive and the repetition for each sample of a batch is therefore the limiting factor for the entire pipeline. This may be less relevant for smaller shapes in the PCPNet benchmark and the sparsely sampled scenes in SceneNN, but for large TLS scans with several million points it is relevant and, in our case, has precluded practical usability.

**Fig. 5:**
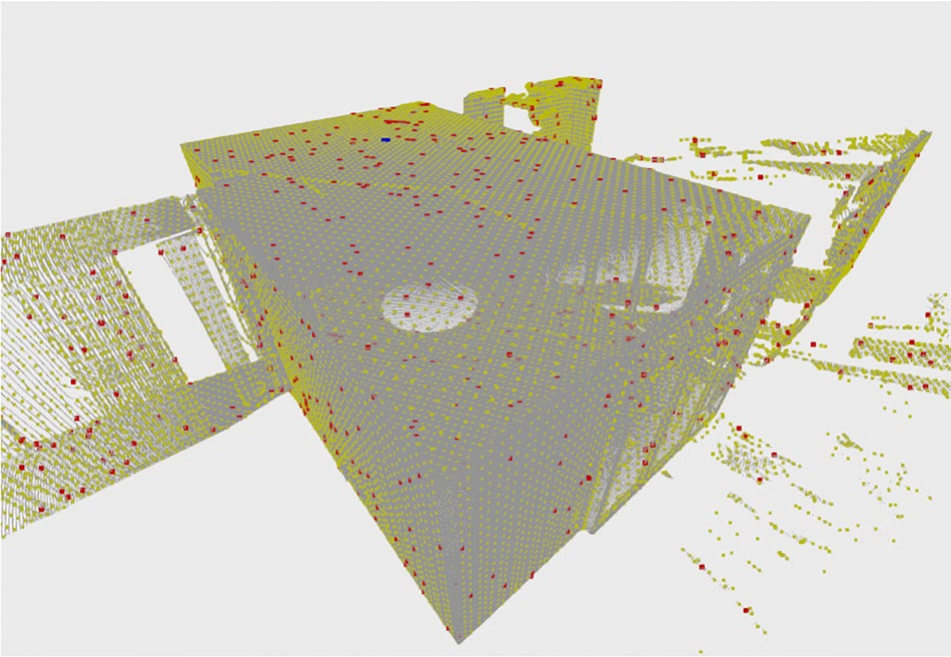
Visualization of the probability sampling to create the global patch. (gray) original input point cloud. (yellow) subsampled voxel grid with fixed voxel size of 10 × 10 × 10 cm. (red) probability-based global patch sample. (blue) query point.

We retained probability sampling to obtain ${{\boldsymbol{P}}}_{q}$, but reduced computational overhead by subsampling ${\mathscr{P}}$, the input for the global patch encoder, using a voxel grid ${\mathscr{P}}$ with fixed voxel size of 10 × 10 × 10 cm for large point clouds. Figure 5 shows the resulting allocation of a point cloud of a normal-sized room. The authors of the original SHS-Net acknowledge that adding some points from uniform sampling can produce better results in structures with different densities and concavities^35^. The unification of the underlying data though discretization prior to the calculation of the probability is consistent with this approach. The discretization with a 10 cm^3^ grid is coarse enough to conserve the global characteristics of the scene and at the same time wide enough for a maximal point cloud reduction.

Consistent alignment of normal vectors across the entire point cloud remains a challenge for large scenes and objects with large holes or disjointed elements. Some^35^ even say that it is completely ambiguous to judge the internal or external orientation of normals of objects and walls inside a room. For the normal features in our dataset, we therefore employ a more stable orientation method, based on the camera viewpoint, to reorient the surface normals after predicting the direction components with SHS-Net. The orientation of all normal vectors towards a fixed point in space provides a solution for the exception that the total point set is captured from a single fixed viewpoint, which is the case with terrestrial laser scanning. Because the viewpoint is static, it can be assumed that no surfaces are facing away from the sensor, and no occluded surfaces exist in the scene. The solution to this problem is trivial if the viewpoint ${v}_{p}$ is known^39^. For all normals ${{\boldsymbol{n}}}_{i}$ to consistently point in the direction of the viewpoint, the following condition must be satisfied:

4 $${{\boldsymbol{n}}}_{i}\cdot {{\boldsymbol{n}}}_{p} > 0$$

5 $${n}_{p}=\frac{{p}_{i}-{v}_{p}}{\Vert {p}_{i}-{v}_{p}\Vert },$$

where ***n***_*i*_ are the predicted surface normal vectors of the point set ***p***_*i*_ and *v*_*p*_ is the camera viewpoint. The normalized vector ***n***_*p*_ between the camera viewpoint and the point set denotes the reference vector for the flipping criterion. All vectors ***n***_*i*_ that do not fulfil the criterion in the Equation (4) will be flipped along their axis.

### Panoramic images

In extent to the point clouds, the dataset includes panoramic point cloud renderings of all 504 scenes covering the full range of the feature spaces (depth, RGB, intensity, and surface normals). These renderings help to visualize the extent of the dataset in a 2D manner, to give the user a quick overview of the scanned areas. The panoramic representation can be further utilized in classic image-processing (2D) computer vision algorithms. The panoramic rendering is achieved by equirectangular projection to map the spherical coordinate space $(\theta ,\phi )$ to a flat image $(u,v)$. In this projection, the longitude and latitude are mapped to horizontal and vertical coordinates of a grid with no transformation or scaling applied. All vertical straight lines remain vertical and straight in an equirectangular projected image, however horizontal straight lines will become curves along the horizon. Poles in this projection are stretched to the entire width of the image at the top and bottom edges^40^. Point clouds are an irregular data format, and empty point cloud regions exist in areas where the sampling density is insufficient, or in places where nothing has reflected the laser. These empty regions are filled with zero-value pixels when mapping into regular pixel space.

## Data Records

The Rohbau3D data records^11^ can be summarized as a medium-scale repository of terrestrial laser scan point clouds covering static scenes from a wide variety of shell construction sides. The records include the spatial coordinates annotated with the sensor-specific (1) RGB color, (2) surface reflection intensity information, (3) the reconstruction of surface normal vectors, (4) panoramic 2D image representations of all feature spaces, and general tools to analyze the data volume.

### Data records overview

The repository contains in total a set of 504 scenes captured in one of 14 different building environments. A textual description of all construction sites, the imaged building types and the content of the scenes is given in Table 1. Six of the 14 construction sites are mid-rise residential buildings, with the conventional subdivision of the property into smaller residential units. Four of the six residential buildings are new apartment blocks that were constructed from the ground up. The other two sites are existing building renovations that conserve the shell of the building after core removal. Three of the ten construction projects are large-sized new school buildings that are further divided into small classroom sections for educational purposes, and one building in the dataset was designed for use as a large, multi-story office building. One site shows the construction of an underground car park and another the vaulted cellar of a historic brick building.

**Table 1:** Index of site-level folders included in the dataset, with neutral observational notes to support navigation and file identification.

File ID	Acquisition Site Overview
site_000	Multi-story apartment block with small to medium-sized rooms in brick wall construction. Some walls plastered, some exposed. Windows present; no doors. Floor mostly dry.
site_001	Multi-story apartment block with small to medium-sized rooms. Sloping ceilings, brick wall construction, walls partially plastered. Windows present; no doors. Floor mostly dry.
site_002	Reinforced concrete underground parking structure with low to high ceilings and column grid. Poor lighting. Water puddles on floor.
site_003	Multi-story school building with large rooms. Reinforced concrete skeleton construction. Good lighting. Water puddles on floor.
site_004	Large hall in reinforced concrete with round ceiling elements. Large floor opening. No facade installed.
site_005	Multi-story school building with rooms of varying sizes. Drywall partitions. Semi-transparent temporary facade covering.
site_006	Multi-story school building with medium to large rooms. Drywall partitions in some areas. Open facade surfaces. Technical equipment installed on ceilings.
site_007	Large hall with high ceiling. Reinforced concrete construction.
site_008	Multi-story office building with small to large rooms and freestanding drywall supports. Glazed facade installed. Technical equipment on ceilings installed.
site_009*	Multi-story brick building under renovation. Historic features. Small rooms and narrow staircases. Windows present; no doors. Poor lighting.
site_010*	Vaulted cellar of brick structure. Small rooms. Uneven floors. Poor lighting.
site_011	Two-story structure with basement. Mixed brick and precast concrete construction. Small to medium rooms. Water on floors. Poor lighting.
site_012	Multi-story apartment block with basement. Reinforced concrete prefabricated construction. Large window and door openings. Some scenes contain water on the floor and show poor lighting.
site_013*	Multi-story brick building under renovation. Small rooms connected by corridors. Walls partly plastered, partly exposed. Mostly clean floors.

Renovation sites are indicated with an asterisk (*).

### The dataset structure

**Fig. 6:**
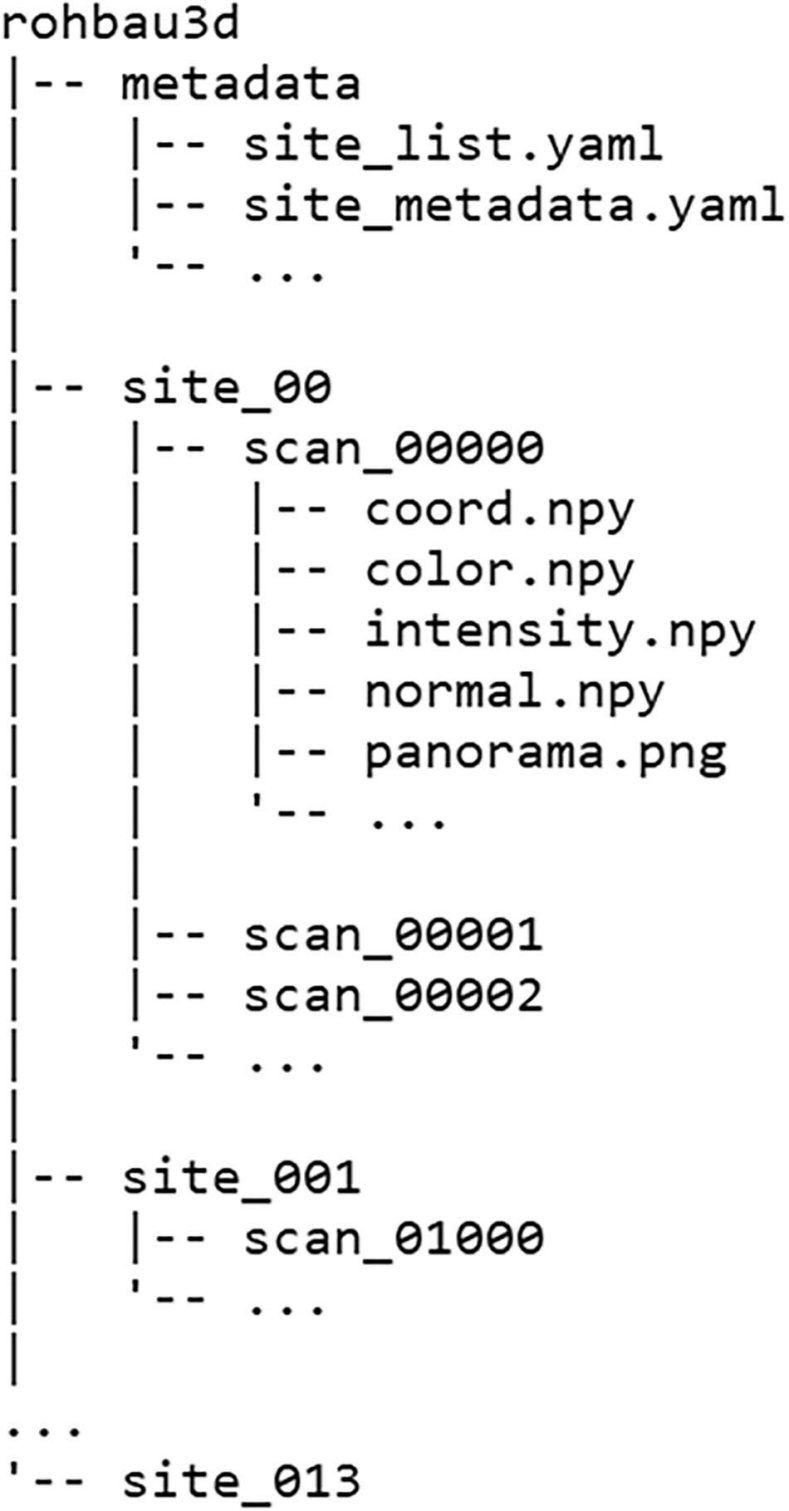
Directory structure of the Rohbau3D dataset^11^.

The data record structure^11^ is organized hierarchically and designed in such a way that it can be expanded at a later date. The folder structure is shown in Fig. 6. The base folder contains all construction site objects with the prefix ‘*site_*’ and a folder for metadata. Each construction site contains an individual folder for each scan object with the prefix ‘*scan_*’. Within a scan object, all data points are saved as separate files. This includes point cloud coordinates, annotations and panoramic images at the time of initial publication. Point cloud features such as color information or surface normal vectors are to be treated like annotations and stored as separate files.

## Technical Validation

The geometric data itself requires comparatively little verification. Pre-processing and filtering of the recordings were deliberately kept to a minimum, as these steps depend on parameter selection and therefore introduce user bias. The goal is to publish sensor data as raw as possible, to reflect the real data output of the device. To validate the overall completeness of the datasets, an objective sanity check was performed. This check verified the presence of all data points within each scene and compared the matrix shapes of the feature vectors to those of the underlying point cloud and a supervised validation database. What is referred to as *smoke testing* in software engineering reveals simple failures in formal data structure and format and is also intended to be run after downloading our dataset to detect corrupted data files. Subjective validation of the data records was performed by a single domain expert during the content moderation. Data curation was initially performed on the 2D panoramic representations of the point clouds and their feature spaces, in order to detect and exclude erroneous scans. In multiple cases, a more in-depth inspection of the data was carried out in 3D space using the open-source software CloudCompare^41^ and Open3D^42^. Scans that did not meet the quality requirements—due to lack of color information, distorted images, or extreme scanning artifacts caused by sudden movement in the scene—were excluded from the dataset. Since we strive for realistic data from construction sites, where movement and disturbances are common, only a few scans were formally excluded.

The quality of surface normal reconstruction is difficult to assess without ground truth values for the specific data. However, a common approach is to test the underlying method using benchmark results from established datasets to infer accuracy when applied to our own data. We compared several state-of-the-art models^35,37,43–45^ to validate the most suitable method for surface normal estimation of unstructured point clouds on two benchmarks.

The PCPNet dataset^37^ consists of a selection of man-made CAD shapes with flat faces and sharp corners, as well as scans of figurines. The benchmark is intended to evaluate local shape properties, such as normal reconstruction. For every shape, 100k points and the corresponding surface orientation are sampled uniformly from the mesh to create a noise-free set. Gaussian noise is applied at three levels of standard deviation (0.12%, 0.6%, 1.2%) to simulate sensor noise, and two point clouds for each mesh are sampled with varying density so that certain regions of the shape are sparsely sampled compared to others. To test the potential real-world performance, we included results from the SceneNN benchmark^14^, which consists of scene reconstructions from RGB-D scans of residential buildings. For the indoor scenes, we only compared the result of unoriented normal estimation, rather than oriented normal, because it is ambiguous to judge the internal or external orientation of normal of objects and walls inside a room where a coherent orientation is user-defined.

The full overview of the test results is provided for unoriented normal estimation in Table 2 and for oriented normal estimation in Table 3. Note that we did not retest all models ourselves; the corresponding test results sources are listed in the ‘source’ column. For comparison, we contrasted the learning-based methods with a classical principal component analysis (PCA) approach, implemented using the Open3D library^42^. The PCA results were highly sensitive to the hyperparameter selection. The results shown in Table 2 represent the optimum from an extended grid search (maximum search radius, maximum number of neighbors). PCA can be used to estimate the vectors, but not their global orientation. In Table 3, we therefore used the propagation-based Open3D function ‘*orient_normals_consistent_tangent_plane*’ with a search radius of k = 30 nearest neighbors, to globally align the vectors.

**Table 2:** Unoriented normal evaluation on PCPNet dataset and SceneNN dataset.

Method	Source	PCPNet Dataset							SceneNN		
		Noise				Density			Clean	Noise	Average
		None	0,12%	0,60%	1,20%	Stripes	Gradient	Average			
PCPNet^37^	^45^	9,64	11,51	18,27	22,84	11,73	13,46	14,58	20,86	21,40	21,13
HSurf-Net^43^	^45^	4,17	8,78	16,25	21,61	4,98	4,86	10,11	7,55	12,23	9,89
GraphFit^44^	^35^	5,21	8,96	16,12	21,71	6,30	5,86	10,69	n.a.	n.a.	n.a.
MSECNet^45^	^45^	3,84	8,74	16,10	**21,05**	**4,34**	4,51	9,76	**6,94**	**11,66**	**9,30**
SHS-Net^35^	^35^	**3,49**	**8,43**	**15,73**	**21,05**	4,45	**4,17**	**9,55**	7,30	n.a.	n.a.
PCA	*	12,48	12,92	18,19	26,96	13,95	13,09	16,27	16,37	16,69	16,53

Reported are the RMSE results under different noise levels and different sampling strategies to simulate sensor noise. Lower is better. The source column declares the resource for the test results. * Indicates our own results.

**Table 3:** Oriented normal evaluation on PCPNet dataset.

Method	Source	PCPNet Dataset						
		Noise				Density		
		None	0,12%	0,60%	1,20%	Stripes	Gradient	Average
PCPNet^37^	^35^	33,34	34,22	40,54	44,46	37,95	35,44	37,66
Hsurf-Net + ODP^43^	^35^	26,91	24,85	35,87	51,75	26,91	20,16	31,07
SHSNet^35^	^35^	**10,28**	**13,23**	**25,40**	**35,51**	**16,40**	**17,92**	**19,79**
PCA	*	23,60	28,66	30,05	41,64	26,00	21,49	28,57

Reported are the RMSE results under different noise levels and different sampling strategies to simulate sensor noise. Lower is better. ODP refers to an orientation with dipol propagation^47^. The source column declares the resource for the test results. * indicates our own validation results.

We applied the same metric as Guerrero *et al*.^37^, Li *et al*.^35^, and Xiu *et al*.^45^ to evaluate the vector deviation error in the estimation. Root mean squared error (RMSE) measures the angle between ground truth normals and predicted normals. RMSE is computed as follows:

6 $${RMSE}=\sqrt{\frac{1}{N}{\sum }_{i=1}^{N}{(\arccos \left(\phi \right))}^{2}\,},$$

where $N$ is the number of evaluated normals in a point cloud, and $\phi$ is the angle cosine of two vectors. In case of unoriented normals, the angle ${\phi }_{u}$ is computed as the absolute inner product.

The quantitative results in Table 2 support the use of learning-based shape understanding for normal estimation over traditional feature-based PCA. The ability of neural networks to adapt and preserve local shape features enables fine-grained reconstruction of the sampled surface gradient, both for the synthetic structures of PCPNet and in the knowledge transfer to the room-scale interiors of SceneNN. The average RMSE in the PCPNet benchmark is very close, with SHS-Net slightly ahead, while MSECNet leads in the room-scale SceneNN benchmark. At the same time, MSECNet can only predict unoriented normals, which is why no test results are provided in Table 3. The PCA results highlight the shortcomings of non-learning-based approaches: the static low-pass filter handles noise reasonably well, but the smoothing of sharp features increases the overall error. The decisive factor for our decision to use SHS-Net for the reconstruction of normals was the lack of an option in MSECNet to consistently orient vectors within the model prediction.

**Fig. 7:**
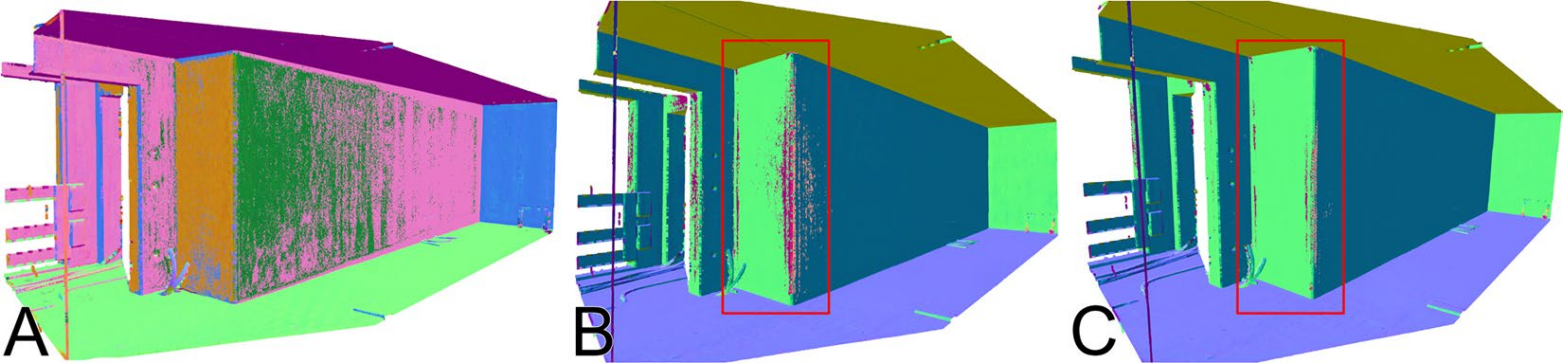
Hallucination in the prediction of oriented surface normal vectors on vertical wall surfaces. (**A**) SHS-Net baseline trained on PCPNet data, without point cloud discretization prior to local and global patch generation. (B) SHS-Net trained on PCPNet data with point cloud discretization prior to patch creation. Voxel size = 1 cm^3^, global patch sample size = 700 nearest neighbors. (C) SHS-Net trained on PCPNet data with point cloud discretization prior to patch creation. Voxel size = 1 cm^3^, global patch sample size = 3200 nearest neighbors.

**Fig. 8:**
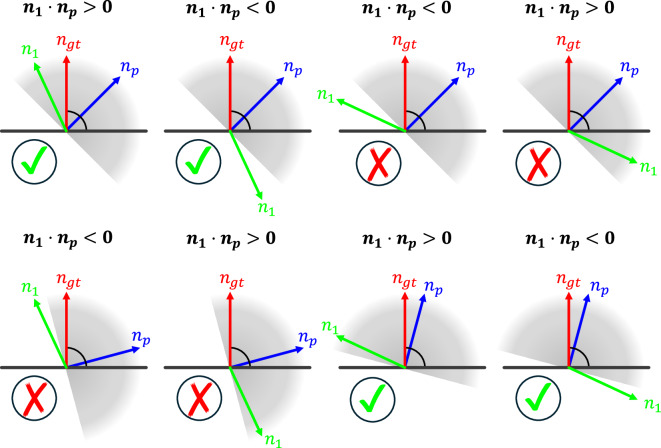
Different cases of flipping ${n}_{1}\cdot {n}_{p} < 0$ or not flipping ${n}_{1}\cdot {n}_{p} > 0$ vector ${n}_{1}$ based on vector ${n}_{1}$. Given the normalized vector ${n}_{p}$ between the viewpoint ${v}_{p}$ and the query point ${p}_{1}$, we propagate its orientation to the vector ${n}_{1}$ based on the vector angle. Note that in cases where the vector ${n}_{1}$ has a large deviation from the ground truth ${n}_{{gt}}$ or the angle between ${n}_{p}$ and the surface is small, false cases exist. The gray hemisphere denotes the angle range, and any vector ${n}_{i}$ within it satisfies the criterion ${n}_{i}\cdot {n}_{p} > 0$. The surface is shown as a black line and its ground truth surface normal vector as a red arrow.

During the validation of the SHS-Net implementation^35^, we found that although the prediction of the vector components (x, y, z) fulfilled the requirements, the consistent assignment of signs to normal vectors pointing inwards or outwards remained a challenge for the construction of interior shapes. The quantitative benchmark results obtained with the learning-based methods (Table 3) are only partially transferable to real production requirements, where reliability is critical. The predicted orientation can hallucinate signs (+/−) under certain conditions, affecting small to large regions. We observed such artifacts mainly in corner regions of vertical wall surfaces, as shown in Fig. 7. Changing the hyperparameters improved the overall results, as indicated in Fig. 7(B,C), where voxelization was introduced prior to patch generation and the size of the sampling neighborhood was increased. However, the results remained unreliable across different scenes. To address this issue, we introduced a normal orientation method based on the camera viewpoint, as described in the Methods section. To consistently orient all normals toward the viewpoint, the condition in Equation (4) must be satisfied. Vectors that do not meet this criterion are flipped along their axis. The different cases for flipping the predicted surface normal vector are visualized in Fig. 8. Note that there are several edge cases in which the criterion falsely corrects the orientation of the vector ${n}_{1}$.

For the technical validation of the results, we considered the possible cases and concluded that the method holds under two soft conditions:

- The estimated normal ${n}_{i}$ must be within a reasonable deviation error $({n}_{{gt}}\cdot {n}_{1})$ of the ground truth normal.
- The angle (${n}_{{gt}}\cdot {n}_{p})$ between the reference vector ${n}_{p}$ and the reflecting surface must not be too small.

In general, the following statement applies: the better the underlying prediction of ${{\boldsymbol{n}}}_{i}$, the more reliably the viewpoint-based criterion can determine the correct sign of the vector. The theoretical threshold for a tolerable prediction error is bounded by the equation:

7 $$\left|{n}_{{gt}}\cdot {n}_{1}\right| < 1-{n}_{{gt}}\cdot {n}_{p}$$

However, since both conditions are mutually dependent and the ground truth normal ${n}_{{gt}}$ is not available in practice, false decisions made by the flipping criterion cannot be further classified. Therefore, the reliability of the criterion decreases in areas far from the camera origin, where the reflection angle becomes smaller and the lower point cloud density leads to less accurate predictions by the model. Limiting the extent of each scan to a sphere with a maximum radius of 25 meters has proven effective in reducing the number of false cases, and thus, errors in the reconstruction of normal features.

**Fig. 9:**
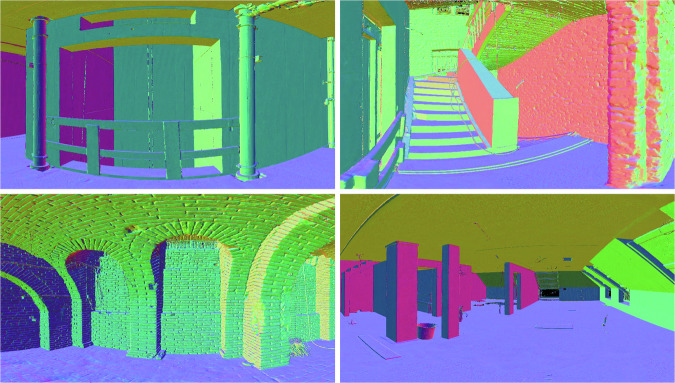
Predicted surface normal vectors for different scenes in the Rohbau3D dataset. The visualized results are produced using a custom implementation of the learning based SHS-Net^35^. The model is trained on the PCPNet^37^ dataset, and the global orientation is unified in post-processing by orienting the normals to the camera viewpoint. The RGB color is derived directly from the components of the normal vector (RGB = (normals + 1)/2) and represents the surface orientation.

While quantitative validation is limited due to the absence of ground-truth normals in the Rohbau3D dataset, we assess the quality of the predicted surface normals through qualitative analysis based on visual coherence, geometric fidelity, and structural consistency. As illustrated in Fig. 9, SHS-Net reliably preserves surface continuity in large planar regions and accurately captures smooth curvatures in architectural elements such as vaulted ceilings, archways, and cylindrical columns. Moreover, the network effectively delineates sharp transitions at the intersections of joined surfaces, e.g. at the edges between two walls or at the corners of rectangular columns. On a more localized level, the predicted normals exhibit sufficient resolution to reflect fine surface structure, such as the textured relief of brick walls or rough wall finishes, which speaks to the model’s ability to represent subtle geometric detail.

To ensure consistent orientation, the predicted normals were globally aligned toward the camera viewpoint during post-processing. This approach proved effective in most scenarios but revealed limitations in regions with low point density, typically farther from the scanner, or in areas affected by sensor noise due to reflective surfaces or by the edge effect^46^ when breaking on the edge of a surface. Despite these localized inconsistencies, the overall quality and structural integrity of the predicted normals suggest they are well-suited for downstream tasks such as segmentation, geometric understanding of indoor scenes, and surface reconstruction.

## Usage Notes

By default, the point clouds are provided for downloading as NumPy data arrays, with each feature stored as a separate vector. This modular structure is designed to facilitate flexible re-use: users can download only the specific features they require, thereby minimizing unnecessary data transfer and memory usage.

More importantly, this design allows the dataset to be easily extended with additional features in the future. For users who prefer working with conventional point cloud formats, we also provide simple conversion scripts that reconstruct standard representations such as PCD and E57 from the NumPy feature vectors.

## Data Availability

All related code to the Rohbau3D dataset is available in our GitHub repository https://github.com/RauchLukas/rohbau3d.
